# Cardioprotective Effects of Methanolic Extract of *Scrophularia frigida *on Ischemia-Reperfusion-Induced Injuries in Isolated Rat Heart

**Published:** 2017

**Authors:** Alireza Garjani, Haleh Vaez, Abbas Delazar, Maryam Rameshrad, Fariba Heshmati Afshar, Parina Asgharian

**Affiliations:** a *Department of * *Pharmacology, * *Faculty of * *Pharmacy, * *Tabriz * *University * *of Medical Sciences, Tabriz, Iran. *; b *Student Research Committee, Faculty of Pharmacy, Tabriz University of Medical Sciences, Tabriz, Iran. *; c *Drug Applied Research Center, Tabriz University of Medical Sciences, Tabriz, Iran. *; d *Department of pharmacognosy, Faculty of Pharmacy, Tabriz University of Medical Sciences, Tabriz, Iran. *; e *Biotechnology research center, Tabriz University of Medical Sciences, Tabriz, Iran.*

**Keywords:** *Scrophularia frigida*, Ischemia, Reperfusion, Arrhythmia, Infarct size

## Abstract

Myocardial infarction is a common presentation of coronary artery disease and the leading cause of death worldwide. The present study investigated potential resistance to ischemia-reperfusion (I/R) injuries following administration of methanolic (MeOH) extract of *Scrophularia frigida* (*S. frigida*) in isolated rat heart. Male Wistar rat hearts (n= 8-10) were isolated and allowed to equilibrate for 20 min, and then subjected to 30 min regional ischemia followed by 120 min reperfusion. In the control group, Krebs-Henseleit (K/H) solution was perfused. However, in the treatment groups K/H solution containing 1, 5, and 10 µg/cc of extract was infused. In addition, total phenol, total flavonoid content and antioxidant property were evaluated and extract was subjected to phytochemical analysis. Administration of extract improved the flow rate, developed pressure as well as max and min left ventricular dp/dt. Number and duration of VT were significantly reduced by all extract concentrations in ischemic phase. In reperfusion phase, significant decreases in single and total arrhythmias were seen. Furthermore, concentrations of 5 and 10 µg/cc of extract remarkably decreased the infarct size. Generally, the methanolic extract of *S. frigida *exhibited a protective effect against I/R-induced injures, which might be due to the antioxidant activiies of iridoids and phenolics.

## Introduction

Myocardial infarction (MI) is one of the most serious ischemic heart diseases that severely affect human health and may lead to long term cardiac complications. Despite improvement in medical and interventional treatment of acute MI, mortality and morbidity rate is still high. The complex mechanisms related to Ischemic/ Reperfusion (I /R) injuries in MI have not completely been known. It is suggested that one of the most important suggested mechanisms is the marked increase in generation of free radicals leading to myocardial damage and unfavorable effects in outcome of acute MI (1, 2). Because of side effects and incomplete efficacy of available chemical drugs on free radical scavenging herbal extracts have been increased. Plants which have some active secondary metabolites with anti-oxidant properties like flavonoids (3, 4), phenyl ethanoides (5), saponins (6, 7), phenyl propanoides (8), phenolic acids (9, 10) and iridoids (11) might be the candidate for investigations on this field. Among these compounds flavonoids and phenylethnaoids as polyphenols have important impact on decreasing cardiovascular diseases (5, 12). One of the plants which is rich in above mentioned secondary metabolites is *Scrophularia *(4, 8), so it is considered to have cardiovascular protective effects on investigation. *Scrophularia* with common Persian name of "gole meimuni" is one of the 220 genus of *Scrophulariaceae* family that consists of 300 species in the world and 42 species in Iranian flora (13).* Scrophularia* species are distributed in the central Europe, Asia, North America and northern hemisphere, speciously along the Mediterranean area (14). Since ancient times, people used some species of this genus for skin disorders, inflammatory conditions and fever as folk remedies (15-17). *S. ningpoensis* is available as a drug in the Chinese pharmacopeia, which has been used to treat upper respiratory disorders, GI difficulties and fever (18). Also radix of *S. ningpoensis* was used for restraining ventricular remodeling in order to ameliorate heart failure with subsequent event of MI (19). *S. frigida* is one of the endemic species in Iran and indigenous in East Azerbaijan, with the common persian name “gole meimuniye yakhchaali. The present study investigated the potential protective effects of aerial parts methanolic extract of* S. frigida* on ischemic/reperfusion induced injuries in isolated rat heart.

## Experimntal

*Animals*

 Healthy male albino Wistar rats (290 ± 20 g) were used in this study. They were housed in standard polypropylene cages, six per cage, under a 12 h light/dark cycle in temperature of

 22 ± 2 °C with 50±10% relative humidity. The animals were given food and water freely. The present study was performed in accordance with the Guide for the Care and Use of Laboratory Animals of Tabriz University of Medical Sciences, Tabriz, Iran (National Institutes of Health Publication No. 85-23, revised 1985). 

*Plant material*

 The aerial parts of *S. frigida* were freshly collected from Mishodagh mountain place located in 15 km of south of Marand in Eastern Azerbaijan province (Iran) in September of 2013. After final botanical identification, the voucher specimen with herbarium number of Tbz-FPh-746 was retained in the School of Pharmacy, Tabriz University of Medical Sciences, Iran. 

*Extraction*

 The fresh aerial parts of *S. frigida* (100 g) were extracted with 1.1 L of n-hexane, dichloromethane (DCM) and MeOH by Soxhlet apparatus. To yield a dry and concentrated extracts, rotary evaporator was used to remove solvents. Among the crude extracts, brownish MeOH extracted sap was kept in refrigerator at 4 °C in sterile screw capped containers until use.

*Phytochemical analysis*

Extracts were tested to identify the existence of active chemical groups such as flavonoids, tannins and iridoids, following standard procedures (16, 22, 23).

*Test for Tannins and phenolic compounds*

 Amount of 5% FeCl_3_ solution was added to tubes of extracts in the presence of tannins. Dark green color was appeared (24). 

*Tests for flavonoids*

*(Shinoda test)*

 Drop wise of concentrated HCL with extract solutions was mixed, then one piece of Magnesium ribbon added which was accelerated the speed of color changes to red.

*Test for iridoids*

1mL of Trim-Hill reagent was added to the concentrated extract and then was heated for a few minutes. A blue-green or red color indicated the presence of iridoids.

*Determination of total phenolic content (TPC)*

 Determination of total phenolic constituents of MeOH extract was evaluated using slight modified Folin- Ciocalteau`s test (21). This method is based on the reducing capacity of Folin- Ciocalteau`s reagent in producing blue color in the samples containing polyphenols. Briefly, 1 mL of prepared extract (5 mg in aqueous acetone 60%) was mixed with 2 mL Folin-ciocalteu reagent and 1 mL of aqueous Na_2_CO_3_. Afterwards the complex mixture was centrifuged in 1200 rpm for 5 min. After incubation at room temperature for 30 min, absorbance of transparent upper mixture was measured at 750 nm using UV spectrophotometer (Pharmacia biotech Ultrospec 2000, UV/Visible spectrophotometer, England) against control (reagent with no extract) for the quantitative phenol estimation. All the process were repeated for different concentrations of gallic acid solution which were previously prepared from 1mg/mL (acetone: water 60:40) of gallic acid stock as a standard. The calibration curve was prepared using the value of absorbance vs. different concentrations. TPC were expressed in terms of gallic acid equivalent (GAE; mg of gallic acid/ g of extract) as an ordinary reference compound.

*Determination of Total flavonoid contents (TFC* *(*

Total flavonoid constituents of the extract was assessed leading a modified assay (25). Concisely, 2 mL of all sample (previously was dissolved in 80% methanol) was mixed with 400 µl of distilled water and 1 mL of AlCl_3_ reagent (133 mg crystalline AlCl_3_ plus 400 mg crystalline sodium acetate in 100 mL of 80% methanol). Thereafter mixtures were allowed to remain at room temperature for 30 min. The absorbance of the reaction mixtures was read at 430 nm vs. blank, spectrophotometrically. Dilutions of quercetin in 5-25 µg/mL of 80% methanol were prepared in the same way and were applied to calculate calibration curve in order to determine the flavonoid quantity. Finally TFC was expressed as quercetin equivalents (mg/ g of extract).

*Determination of Free-radical-scavenging activity in-vitro*

 Antioxidant activity of the extract was assessed using the 2, 2-diphenyl-1-picrylhydrazyl (DPPH) obtained from Sigma Aldrich Company. The DPPH assay was carried out as described by Takao *et al* (20). Stock solutions of the extract was prepared as 1 mg/mL in MeOH .Serial dilutions were made to obtain concentrations of 5×10^-1^, 2.5×10^-1^, 1.25×10^-1^, 6.25×10^-2^, 3.13×10^-2^ and 1.56×10^-2 ^mg/ml. Diluted solutions of extract (5 mL each) were mixed with 0.08 mg/mL DPPH solution (5 mL) and allowed to stand for 30 min for occurring any reaction. The UV absorbance was recorded at 517 nm. The experiment was done in triplicate and the inhibition percent of free radical DPPH in percent (I %) was calculated in the following way:

I% = (A _blank_ – A _sapmle_)/ A _blank_ × 100

Where A _blank_ is the absorbance of the negative control (containing all the reagents except the extract), and A _sapmle_ is the absorbance of the test samples. Extract concentration providing 50% inhibition (IC_50_) was calculated. Quercetin was used as positive control.

*Preparation of isolated heart perfusion*

 Preparation of isolated heart perfusion was performed as previously described (26) with minor modifications. Male Wistar rats were heparinized (1000 IU/kg; i.p.) and then anesthetized with ketamine/xylasin (60 / 10 mg/kg; i.p.). When the rats didn’t responded to external stimuli, the surgery for harvesting the heart was done. Harvested heart was transferred as soon as possible to a dish containing ice cold modified Krebs–Henseleit buffer (K/H) and mounted immediately to the langendorff apparatus (ML176-V Langendorff Apparatus, ADInstruments, Australia). The hearts were perfused at a constant pressure (80 mmHg) with a K/H containing NaCl 125, KCl 4.3, KH_2_PO_4_ 1.1, MgCl_2_. 6H_2_O 1.3, CaCl_2_. 2 H_2_O 2.4, NaHCO_3_ 25, and glucose 13.32(in mmol/l). The perfusate was gassed with carbogen (5% CO_2_/95% O_2_) to set the pH in 7.38-7.56 at 37°C. 

When the harvested heart was mounted to the apparatus, the suture by 6.0 silk surgical is put in place around the left anterior descending artery (LAD) and formed to make a snare. During ischemia, the snare is tightened around LAD and loosed during reperfusion. To measure left ventricular contractility, a latex balloon attached to a pressure transducer (MLT844 physiological pressure, ADInstruments, Australia) was inserted into the left ventricular cavity via the mitral valve after removing the atrial appendage.

After stabilization period (15 min) with infusion by K/H, time was set to zero and K/H without or with extract (1, 5, and 10 µg/cc in separate groups) was infused 5 min before occlusion and maintained for duration of the experiment.

*Measurement of myocardial infarct size*

 According to Bell *et al* (26) study with some modification, to determine the infarct size double staining strategy was used. At the end of 120 min reperfusion period, the ligature around the LAD artery re-tied. The cannulated heart was detached and perfused slowly by 1 mL Evans blue dye (0.25% w/v) via aortic cannula. Then the heart was stored at -20 °C. For second staining the frozen heart was sliced from apex to base (into 1-2 mm sliced). The slices incubated with 1% (w/v) triphenyltetrazolium chloride (TTC) solution in phosphate buffer for 15 min at 37 °C to dye the non–infarcted region. At the end, the slices were fixed in 10% formalin overnight. This procedure resulted in the normally perfused tissue being stained blue, non-infarcted and non-perfused tissue stained brick red, infarcted tissue remaining unstained and appeared pale. Digitally photographed sliced were imported to Image J software (Wayne Rasband, National Institute of Health, USA) and infarct size was computed.

*Statistical analysis*

 Except for the incidence of ventricular tachycardia (VT) and ventricular fibrillation (VF) that indicated as percentage, all results expressed as mean± SEM. To compare the number of VT, ventricular ectopic beats (VEBs), duration of VT and VF between groups and the percentage of infarct size and all hemodynamic factors, the Mann-Whitney non-parametric U-test were employed. Analyzing the incidence of VT and VF was accomplished by Fisher test. Differences were considered significant at a level of *P*< 0.05.

## Results

*Phytochemical results*

 Preliminary phytochemical trials of MeOH extract of *S. frigida* illustrated the existence of iridoids, phenolic compounds (tannins) and flavonoids.

*TPC and TFC results*

 The total amount of phenolic compounds was 25.67 ± 0.07 mg GAE/g of extract and the constituent of flavonoids was equal to 72.08 ± 1.63 mg rutoside equivalent in 1 g of powdered plant material.

*Free-Radical-Scavenging Activity*

 Free radical scavenging activity of the MeOH extract was based on the reduction of DPPH. The potency of antioxidant activity of the extract (IC_50_) was (0.134 ± 0.04mg/ml) in comparison to the value of quercetin (0.003 ± 0.00 mg/ml) as a positive control.

*Effects of* *MeOH extract of S. frigida* *on ischemia/reperfusion heart model*

*Flow rate*

**Figure 1:**
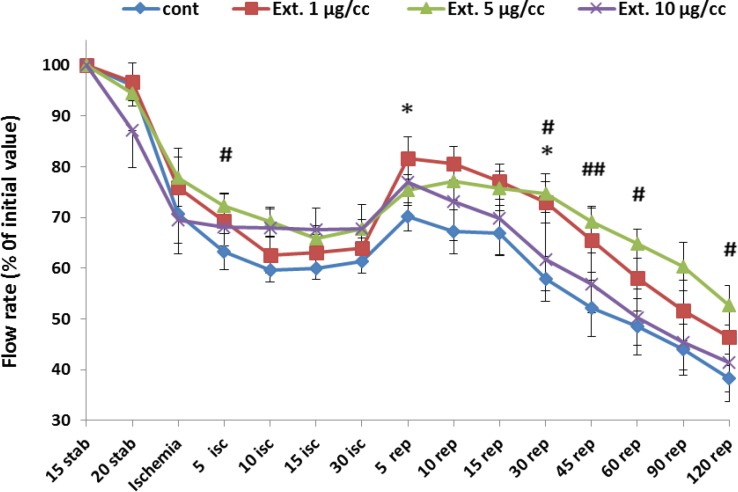
Perfusion rate in the control and treated groups receiving 1, 5 and 10 µg/cc of the extract during whole experiment period. Data are represented as Mean±SEM. Stab: stabilization, isc: ischemia, rep: reperfusion, cont: control, Ext: extract. ^*^p<0.05 significant difference of 1 µg/ml extract group from control group. ^#^p<0.05 and ^##^p<0.01 significant difference of 5 µg/cc extract group from control group. N=8-10 rats in each group

Figure 1 represents the change of flow rate (% of initial value) during whole period of experiment. Ligation of coronary artery at the end of stabilization led a marked decline in perfusion flow rate from 95% to 63%. Reperfusion of the ischemic area again caused an increase in the flow rate to 70% that gradualy decreased to 38% at the end of the reperfusion time. The extract at the concentration of 1 µg/cc improved the flow rate significantly in the 5 and 30 min of reperfusion from 70 and 57 in control group to 81 and 72, respectively (p<0.05).Furthermore, the best improvement of perfusion rate was shown at 5 µg/cc of the extract. The increase in perfusion rate was significant in 5 min of ischemia and 30, 45, 60 and 120 min of reperfusion compared to control group ( p<0.05 for all mentioned times except 45 min of reperfusion with p< 0.01). 

*Heart rate*

**Figure 2:**
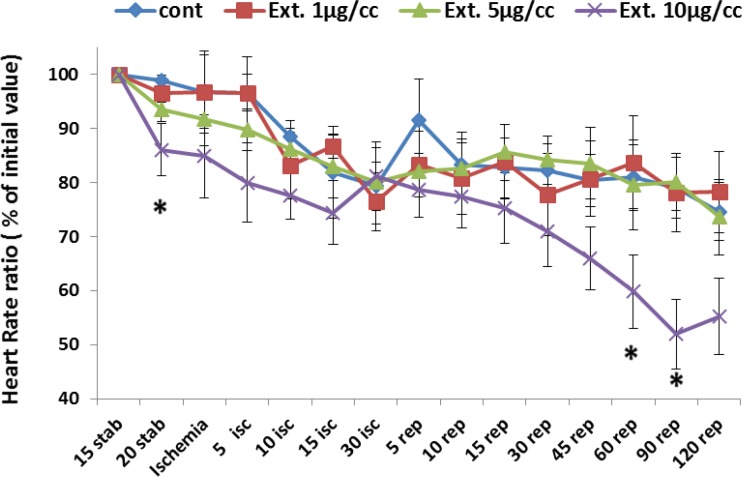
Heart rate in the control and treated groups receiving 1, 5 and 10 µg/cc of the extract during whole experiment period. Data are represented as Mean±SEM. Stab: stabilization, isc: ischemia, rep: reperfusion, cont: control, Ext: extract. . ^*^p<0.05 significant difference from control group. N=8-10 rats in each group

Figure 2 represents the change of heart rate (% of initial value) during whole period of experiment. Ligation of coronary artery at the end of stabilization led a continious decline in heart rate from 98% to 79% at the end of ischemic phase. With reperfusion of the ischemic area an increase occurred in the heart rate by 85% that gradualy decreased to 74% at the end of the reperfusion time. The heart rate changes under perfusion of extracts at 1 and 5 µg/cc was similar to that of the control group. However, the extract at the concentration of 10 µg/cc caused bradycardia which reached a significant level (p<0.05) at 20 min of stabilization and 60 and 90 of reperfusion time.

*Developed pressure*

**Figure 3:**
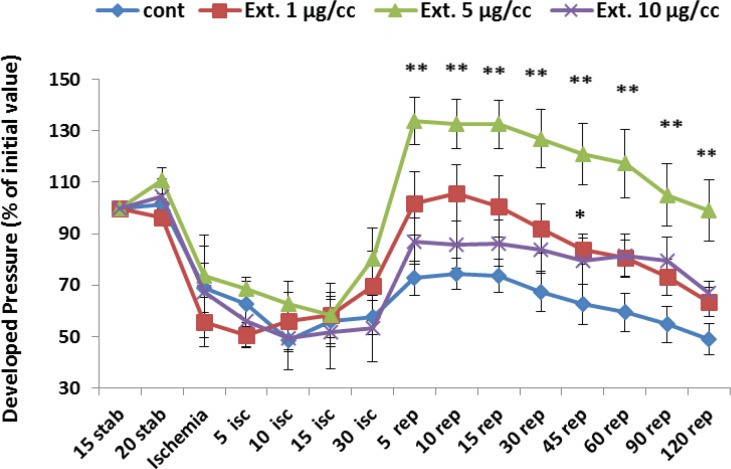
Developed pressure percent in the control and treated groups receiving 1, 5 and 10 µg/cc of the extract during whole experiment period. Data are represented as Mean±SEM. Stab: stabilization, isc: ischemia, rep: reperfusion, cont: control, Ext: extract. ^*^p<0.05 and ^**^p< 0.05 significant difference from control group. N=8-10 rats in each group.

Figure 3 represents the developed pressure changes (% of the initial value) during whole period of experiment which is determined as the difference of systolic and diastolic pressure. Ligation of coronary artery at the end of stabilization led a marked decline in developed pressure from 100% to 62%. Reperfusion of the ischemic area caused an increase in the developed pressure to 72% of the intial value that graduatly decreased to 49% at the end of the reperfusion time. The extract at the concentration of 1 µg/cc significantly improved the developed pressure during first 45 min of reperfusion time (p<0.05). Furthermore, the best improvement of developed pressure was seen by 5 µg/cc of the extract, which kept the pressure at a high level (p<0.01) during whole period of reperfusion.

*Maximum and minimum Left Ventricular dp/dt*

**Figure 4:**
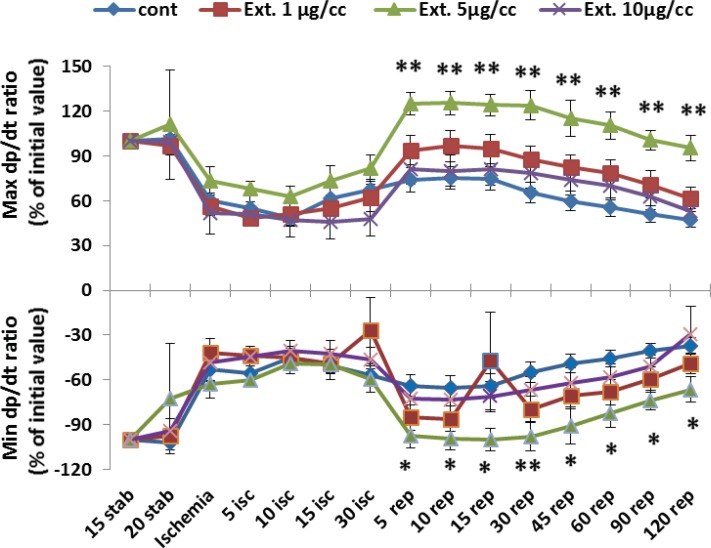
Max and Min LV dp/dt ratio percent in the control and treated groups receiving 1, 5 and 10 µg/cc of the extract from 15 min during experiment. Data are represented as Mean±SEM. Stab: stabilization, isc: ischemia, rep: reperfusion. *p< 0.05 and **p< 0.05 significant difference between 5 mg/kg Ext. group and control group. N=8-10 rats in each group

 The result of changes of maximum and minimum of LV dp/dt during perfusion time are shown in figure 4. There are no significant difference in the changes of max and min LV dp/dt in group receiving 1 and 10 μg/cc of extract in comparison to control group. However, as like as developed pressure, in concentration of 5 μg/cc of extract there are considerable difference in all reperfusion times compared to control group (p<0.01).

*Effects of* *MeOH extract of S. frigida* *on ischemia/reperfusion-induced arrhythmia in isolated rat heart*

 The effects of administration of extract on reperfusion-induced cardiac arrhythmias after 30 min regional ischemia are summarized in table 1. Perfusion of the isolated heart with 10 µg/cc of extract significantly decreased the number of single arrhythmias in ischemic phase (p<0.05). Furthermore, the number of ischemic total arrhythmias was also significantly decreased by 5 and 10 µg/cc of the extract (p<0.05).

The number of ischemic VT and the duration of VT were significantly reduced by all concentration of the extract (p<0.05 in 1 and 10 µg/cc and p<0.01 in 5 µg/cc). The incidence of VT and VF were decreased with all three concentration of the extract, but the reduction did not attain a significant level (Table 1).

**Table 1:** Effects of MeOH extract of *S. frigida* (1, 5 and 10 µg/ml) on I/R-induced cardiac arrhythmias after 30 min regional ischemia followed by reperfusion in isolated rat heart

**Ischemic phase**	**Number**					**Duration (sec)**		**Frequency (%)**	
	Single	Salvous	Triplet	VT	Total arrhythmias	VT	VF	VT	VF
Control	354 ± 58	24 ± 4	11 ± 2	894 ± 190	1285 ± 246	122 ± 26	20 ± 12	100	50
Ext. 1 mg/l	301 ± 42	26 ± 5	17 ± 3	215 ± 96*	560 ± 112	23 ± 9*	24 ± 17	100	66.7
Ext. 5 mg/l	235 ± 41	19 ± 4	23 ± 7	166 ± 55**	444 ± 91*	23 ± 7**	7 ± 3	85.7	42.9
Ext. 10 mg/l	143 ± 34*	16 ± 5	9 ± 3	261 ± 112*	430 ± 152*	34 ± 14*	39± 39	66.7	16.7
**Reperfusion phase**	**Number**					**Duration (sec)**		**Frequency (%)**	
	Single	Salvous	Triplet	VT	Total arrhythmias	VT	VF	VT	VF
Control	141 ± 24	10.1 ± 2.7	1.8 ± 0.7	224 ± 109	377 ± 119	30 ± 15	2.9 ± 2.4	75	25
Ext. 1 mg/l	51 ± 9**	3 ± 1.1*	2.3 ± 1.1	11.5 ± 7.3	67 ± 17*	1.5 ± 1	0.7 ± 0.7	33.3	16.7
Ext. 5 mg/l	54 ± 20**	2.8 ± 1*	1.4 ± 0.4	3.7 ± 1.5*	68 ± 24**	0.6 ± 0.4	0	28.6	0
Ext. 10 mg/l	85 ± 10*	4.8 ± 1.8	1 ± 0.4	9.3 ± 9.3	95 ± 20*	1.3 ± 1.3	7.5 ± 7.5	16.7	16.7

* p<0.05 and

** p<0.01 *versus *the control group. N= 8-10 in each group. Cont: control, Ext: extract, VT: Ventricular Tachycardia, VF: Ventricular Fibrillation, Total arrhythmias: Single+ Salvos+ Triplet+ VT

In the reperfusion phase, the number of single and the total number of arrhythmias were decreased significantly by all three concentration of the extract (p<0.05 in 1 and 10 µg/cc and p<0.01 in 5 µg/cc). Also the number of salvous arrhythmias reduced significantly with 1 and 5 µg/cc of extract (p<0.05). In addition, concentration of 5 µg/cc significantly reduced the number of VT in comparison to control group (p<0.05). In contrast to ischemic phase, there was no significant difference of reductions of VT duration and incidence in extract compared to control group. The duration of VF in both ischemic and reperfusion phase did not change in a significant manner in comparison to control group. 

*Effects of MeOH extract of S. frigida on infarct size in the isolated rat heart*

 As demonstrated in Figure 1, the infarct size was 66.25±2.8% in the control group while the perfusion of the MeOH extract of *S. frigida* by 5 and 10 µg/cc remarkably decreased it to 41.28±1.3% and 17.88±1.35% (*P*<0.001), respectively. 

**Figure 5:**
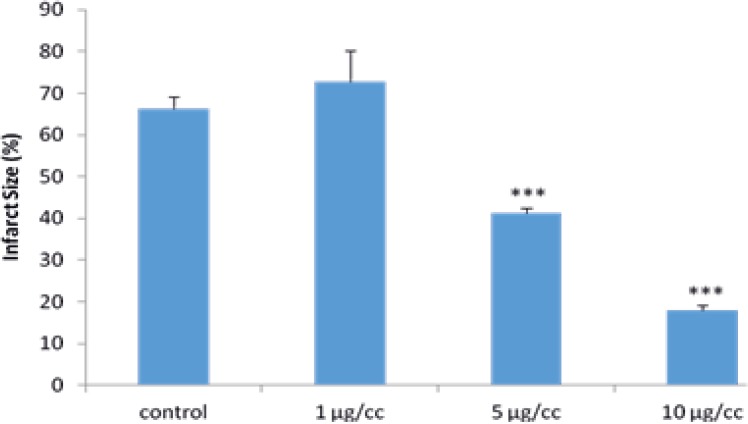
Myocardial infarct size in the control and isolated rat hearts receiving methanol extract of *S. frigida* (1, 5, 10 µg/cc) during 30 min ischemia followed by 120 min reperfusion. Data are represented as mean±SEM. ***p<0.001 versus control group respectively. N average = 5 rats in each group

In addition, the 1 µg/cc of extract had no significant effect on the infarct size following 30 min ischemia and 2 h reperfusion (Figure 5). 

## Discussion

 Myocardial infarction is the most important cause of mortality that makes a threat for human health (27). As far as we know, ischemia followed by reperfusion causes pivotal damages to myocardium due to striking increase in generation of free radicals (1). Owing to the fact that antioxidants restrain oxidative damage, it is clear that flavonoids and polyphenols have an important effect on decreasing CVDs (5, 12). 

Genus *Scrophularia* which is contained high amount of iridoids, polyphenols and flavonoids (9, 10, 28, 29) is being used traditionally for treatment of inflammatory disorders (15-17). However, little is known about cardio protective actions of *Scrophularia *species in different cardiovascular diseases. In the present study, we investigated antioxidant, TPC and TFC activity of the MeOH extract of* S. frigida* aerial parts along with its therapeutic efficacy on ischemia/reperfusion induced arrhythmias and on infarct size in the isolated rat heart.

The effect of MeOH extract on hemodynamic factors demonstrated that the extract had high amounts of TPC, TFC, and good antioxidant activity which cause an improvement on perfusion rate and contractility of isolated rat hearts subjected to ischemia and reperfusion. A considerable cardio protective effect against ischemia/reperfusion induced injury by increasing coronary perfusion and left ventricular developed pressure (LVDP) as well as by reversing the myocardial depression through improvement of cardiac contractility and relaxation. The administration of extract during 15 min of stabilization, resulted a decrease in heart rate in all groups, which was significant by 10 µg/cc. Decreased number of single arrhythmia that was recorded in 10 µg/cc of extract may be related to bradycardia induced by high extract concentration.

VT and VF induced by spontaneous restoration of perfusion remain the most important causes of sudden death following reperfusion (30-32). The mechanisms involved in I/R-induced arrhythmias may include heterogeneous recovery of conduction and a refractory period of incomplete reperfusion, reentry, abnormal automaticity and activities triggered by Ca^2+ ^overload and free radicals. Wide range of antiarrhythmic drugs are available, however, have limitations arising from their toxic and pro-arrhythmic potential. Therefore, development of new agents for I/R-induced arrhythmias is severely required. In this study, perfusion of K/H solution containing *S. frigida *extract reduced the number and duration of VT in ischemia period in all concentrations. In reperfusion phase, despite the decrease in single, salvous and total arrhythmias in all extract concentrations, the significant decrease in VT number just exhibited by 5 µg/cc of extract. The interpretation of these results may be related to the different proportion of various compositions in each extract concentration. The result obtained by infarct size assessment test, confirmed the effective role of *S. frigida *extract in reducing I/R-induced damages and cell death in dose of 5 and 10 µg/cc. 

Numerous species of the *Scrophularia* have been studied phytochemically revealing the presence of biologically active polyphenolics, flavonoids, iridoids and terpenoids (33-35). Also anti-inflammatory and cardiovascular effects of mentioned secondery methabolites were proved previously in other plants (36-42). Furthermore, some compounds such as anethole, anisaldehyde, eugenol, benzaldehyde, eugenol acetate are common in the *Scrophularia*. On the other hand, the presence of aromatic compounds in various genera of the *Scrophulariaceae *is one of the characteristics of this family (43). Among these components, agents with antioxidant activity (aromatic and phenolic compounds) play the principle role in prevention and treatment of ischemic heart injuries especially arrhythmias. Furthermore, presence of compounds like glycosides can improve cardiac contractility and cell feeding in both ischemic and reperfused conditions. Moreover, the effect of methanolic extract of other species of this genus on Ischemia/Reperfusion in isolated rat heart has not been studied. However, the other investigations have evaluated the cardio protective effect of the plants on ischemia-reperfusion-induced injuries in isolated rat heart (44-46). In comparison to the results of mentioned studies, *S. frigida *showed the significant effect on lower doses which it seems to be considered as a good target for the future investigation on natural cardio protective products. We suggest that the cardio protective effect of different fractions of methanolic extract of *S. frigida *to investigate, then effective fractions analyze to find the major effective pure compounds in order to comparing with commercial available cardio protective drugs. 

## Conclusion

In conclusion, this study demonstrates that the treatment of isolated hearts with MeOH extract from aerial parts of *Scrophularia frigida* caused a pronounced reduction in myocardial dysfunction and damages arising from regional ischemia (30 min) and reperfusion (120 min). The extract may contain different constituents which act as protective agents in dose-dependent manner. We found that, 5μg/cc has showed better cardio protective effect than 1 and 10 μg/cc. Since IR models of rat isolated heart mimic clinical myocardial infarction or intracoronary thrombolysis situations, it is expected that with further research on components and their mechanism and clinical studies, *S. frigida *extract may become effective antiarrhythmic drugs. 
